# Closed-loop control of spinal cord stimulation to restore hand function after paralysis

**DOI:** 10.3389/fnins.2014.00087

**Published:** 2014-05-19

**Authors:** Jonas B. Zimmermann, Andrew Jackson

**Affiliations:** ^1^Faculty of Medical Sciences, Institute of Neuroscience, Newcastle UniversityNewcastle Upon Tyne, UK; ^2^Donoghue Lab, Department of Neuroscience, Brown UniversityProvidence, RI, USA

**Keywords:** intraspinal microstimulation, closed-loop neuro-prosthetics devices, spinal cord injury, brain-computer interface, hand movements, grasp, electrical stimulation, non-human primate model

## Abstract

As yet, no cure exists for upper-limb paralysis resulting from the damage to motor pathways after spinal cord injury or stroke. Recently, neural activity from the motor cortex of paralyzed individuals has been used to control the movements of a robot arm but restoring function to patients' actual limbs remains a considerable challenge. Previously we have shown that electrical stimulation of the cervical spinal cord in anesthetized monkeys can elicit functional upper-limb movements like reaching and grasping. Here we show that stimulation can be controlled using cortical activity in awake animals to bypass disruption of the corticospinal system, restoring their ability to perform a simple upper-limb task. Monkeys were trained to grasp and pull a spring-loaded handle. After temporary paralysis of the hand was induced by reversible inactivation of primary motor cortex using muscimol, grasp-related single-unit activity from the ventral premotor cortex was converted into stimulation patterns delivered in real-time to the cervical spinal gray matter. During periods of closed-loop stimulation, task-modulated electromyogram, movement amplitude, and task success rate were improved relative to interleaved control periods without stimulation. In some sessions, single motor unit activity from weakly active muscles was also used successfully to control stimulation. These results are the first use of a neural prosthesis to improve the hand function of primates after motor cortex disruption, and demonstrate the potential for closed-loop cortical control of spinal cord stimulation to reanimate paralyzed limbs.

## Introduction

A long-standing ambition of neural prosthetics has been to reconnect artificially parts of the nervous system that have been disconnected as a result of injury (Craggs, 1975). One such application is the treatment of paralysis after a spinal cord injury or stroke that disrupts the pathway by which volitional motor intent encoded in premotor areas is relayed via corticospinal projections from M1 to motorneurons in the spinal cord (Lemon, 2008). In the absence of descending input, electrical stimulation of spinal motor circuits can generate functional movements including walking in the lower-limb (Mushahwar et al., 2000) and grasping in the upper-limb (Moritz et al., 2007; Zimmermann et al., 2011). Epidural stimulation of the spinal cord has recently been used to facilitate standing and walking in a spinal cord injured patient (Harkema et al., 2011), but thus far clinical applications have used pre-set trains of stimuli delivered in an open-loop mode.

Brain-Machine Interface techniques developed first in monkeys (Wessberg et al., 2000; Serruya et al., 2002; Velliste et al., 2008) and now translated to human patients use control signals extracted from cortical spiking activity to operate assistive devices including computer cursors (Hochberg et al., 2006), robotic prostheses (Hochberg et al., 2012; Collinger et al., 2013), and functional electrical stimulation of muscles (Moritz et al., 2008; Pohlmeyer et al., 2009; Ethier et al., 2012). A case study in one monkey with upper-limb paresis used cortical local field potential activity to control intraspinal stimulation and restore the ability to generate isometric torque with the wrist (Nishimura et al., 2013a).

Here we tested whether a neural prosthesis could be used to restore volitional grasping in two monkeys following reversible inactivation of the primary motor cortex (M1) using muscimol. Spike activity recorded from ventral premotor cortex (PMv) or residual electromyogram (EMG) activity was used to control stimulation of the spinal cord.

## Materials and methods

Experiments were approved by the local ethics committee at Newcastle University and appropriate UK Home Office licenses in accordance with the Animals (Scientific Procedures) Act 1986.

### Behavioral task

**Figure 1:**
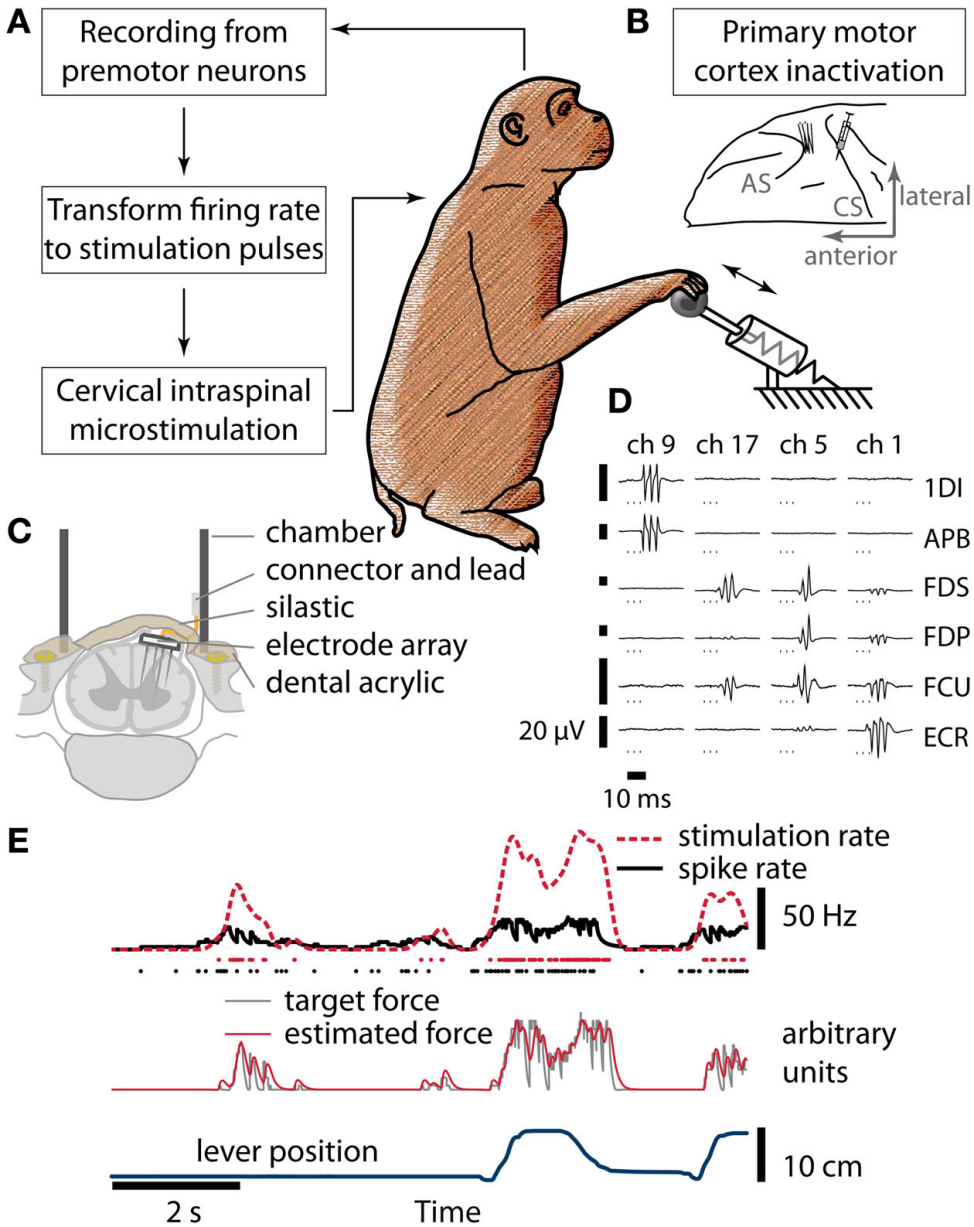
**Brain control of spinal cord stimulation after reversible paralysis. (A)** Schematic of the closed-loop stimulation protocol. Spiking activity recorded from neurons in PMv was converted in real-time to stimulation delivered to the spinal cord while monkeys performed a grasp-and-pull task. **(B)** Hand and forearm muscles were temporarily paralyzed by micro-injections of muscimol into hand area of M1, anterior to the central sulcus (CS). Neural activity was recorded from PMv, in the posterior bank of the arcuate sulcus (AS). **(C)** Cross-section of the spinal stimulation implant used in monkey R. **(D)** Sample EMG responses elicited by stimulation of different channels of the FMA (arranged in columns, channels 1, 5, 9, and 17 of the array chosen to illustrate different response properties; 3 biphasic pulses at 300 Hz, stimulation currents between 12 and 81 μA. 1DI, first *dorsal interosseus*; APB, *abductor pollicis brevis*; FDS, *flexor digitorum superficialis*; FDP, *flexor digitorum profundus*; FCU, *flexor carpi ulnaris*; ECR, *extensor carpi radialis*). **(E)** From neural spikes to stimulation pulses (simulated data). Spikes (black dots) were used to estimate instantaneous firing rate (black line) in real time. The firing rate was transformed to yield a target force function (gray line, arbitrary units). Whenever the estimated stimulation-induced force (red line, a. u.) was below the target, a stimulus pulse (red dots) was delivered. Smoothed stimulation rate (red broken line) and lever position (blue line) are shown for comparison.

Two female, purpose bred and food-restricted Rhesus macaque monkeys *(Macaca mulatta)* were trained to perform a self-paced reach-and-grasp task (Figure 1A). Different handles (squash ball, horizontal bar) were attached to a spring-loaded lever (spring 1: initial force 1.6 N, spring constant: 120 N/m; spring 2: 0.8 N, 50 N/m; spring 3: 0.4 N, 20 N/m). Monkeys were required to pull and hold the lever at a minimum target displacement (0.5–5 cm) and for a set time (0.5–1 s) chosen to accommodate the monkeys' level of paralysis and motivation. After successful trials, monkeys were required to return the lever to the initial position and remain there for 3 s. Different tones indicated when the lever entered the target position, the end of the hold time, and the end of the home waiting period. For every successful trial, monkeys were rewarded with a small piece of fresh or dried fruit or a drop of yoghurt. While the parameters varied between sessions, they were kept constant within a session after an initial adjustment period.

### Surgical procedures

Three implant surgeries were performed per monkey, each separated by about 1 month. In the first surgery, six electromyogram (EMG) patch electrodes were implanted over hand and forearm muscles and tunneled subcutaneously to a connector on the head (1DI, first *dorsal interosseus*; APB, *abductor pollicis brevis*; FDS, *flexor digitorum superficialis*; FDP, *flexor digitorum profundus*; FCU, *flexor carpi ulnaris*; ECR, *extensor carpi radialis*). The wrist extensor ECR was chosen as a control to monitor paralysis and stimulation effects. In addition, a recording chamber was fixed over the right arcuate sulcus for intracortical microstimulation (ICMS) mapping. The hand region of PMv was identified from low threshold (<50 μA) responses in hand muscles to ICMS trains (13 or 20 biphasic pulses, 200 μs per phase, at 300 Hz, delivered during mapping sessions under ketamine/medetomidine sedation). In a second surgery, a custom-made moveable microwire array (Jackson and Fetz, 2007; 12 microwires in monkey B, 15 microwires in monkey R) was implanted at the PMv target location and a chamber was fixed over M1. In the third surgery, a laminectomy of vertebrae C5-C7 was performed. A percutaneous recording chamber was fixed using dental acrylic and titanium screws inserted into the lateral masses of vertebrae C4-T1 (Perlmutter et al., 1998). In monkey B, we used a grid inserted into this chamber and a miniature screw microdrive (MO-903B, Narishige, Japan) to insert electrodes (Pt/Ir, 125 μm diameter, Microprobes Inc., Gaithersburg, MD) acutely into the spinal cord during experimental sessions. In monkey R, we made a midline dural incision and inserted a custom Floating Microelectrode Array (Microprobes Inc.) for chronic stimulation of motorneuron pools (17 electrodes, lengths 3–5 mm, 50 kΩ nominal impedance, Figure 1C). The dura was then closed with sutures and sealed with 2-part silastic (Kwik-Sil, WPI Inc., Sarasota, FL). The connector was mounted inside the chamber with dental cement.

### Pharmacological inactivation

The hand area of M1 was identified by low threshold ICMS (<20 μA, 13 pulses at 300 Hz) responses in hand muscles. Muscimol (Sigma-Aldrich, M1523 dissolved in sterile saline to 0.5%) was injected into hand area of M1 (Figure 1B) under ketamine/medetomidine sedation before stimulation experiments using a 2.5 μl Hamilton syringe and a 31-gauge needle. Injections were delivered at 3 depths per site (5, 3.5, and 2 mm below dura), 0.5 μl per depth injected slowly over 30 s. In monkey R, up to 3 injection sites and 4 depths were used. Once the injections were complete, the sedation was reversed using atipamezole to ensure the monkeys were sufficiently alert (usually within 30 min) to perform the task while the muscimol inactivation was still in effect.

### Intraspinal microstimulation protocol

At the beginning of each experimental session, we assessed the stimulation thresholds of the intraspinal electrodes. We used trains of 3 biphasic pulses (200 μs per phase, 300 Hz) and increased the current up to 100 μA until we either observed movements of arm or hand muscles or stimulus-evoked potentials in any of the EMG recordings in at least 50 percent of the cases. For subsequent closed-loop stimulation, we generally chose an electrode whose stimulation caused wrist or finger flexion movements. In monkey R, where multiple electrodes were available simultaneously, the electrode evoking strongest movements at low amplitudes (~50 μA) was chosen.

We recorded action potentials of premotor neurons (1401, CED, Cambridge, UK; 1–15 units discriminated in real-time using a template-matching algorithm implemented in Spike2 software, CED). The neuron whose firing correlated best with onset of movement in the previous, non-stimulation session was chosen to drive ISMS. If that neuron was not available, the animal was observed attempting the behavioral task and a task-related neuron was chosen based on its evident activity pattern, i.e., consistent modulation of firing frequency during several consecutive trials. Discriminated spike events were transmitted in real time to a computer that estimated the instantaneous firing rate (Figure 1E). The firing rate was transformed (shifted, scaled, delayed) and this transformed rate was used as a target force function for stimulation (Zimmermann et al., 2011). This target force was then used to estimate when stimulation pulses had to be delivered in order to best match the force. We assumed that each pulse results in a force response corresponding to a critically damped system (Milner-Brown et al., 1973) with a time constant of 50 ms. Thus, muscle force was modulated by changing the stimulation frequency. Shift and scale of the neural firing rate were chosen to maximize the dynamical range of spinal stimulation frequency. Stimulation frequency was limited to 125 Hz based on our previous finding that trains of up to 100–125 Hz are sufficient to elicit tonic contractions of hand and arm muscles (Zimmermann et al., 2011 and unpublished data). Usually, task-related premotor neurons we recorded assumed their maximum (or minimum) firing rate 100–250 ms before movement onset. Therefore, a delay was chosen such that the maximum stimulation frequency coincided with the monkey's attempted movement. Due to a constant processing delay due to online spike discrimination and firing rate estimation the additional delay was in the order of 100–200 ms. Stimulus pulses (biphasic, cathodic first, 200 μs per phase, 10–200 μA) were delivered to one intraspinal electrode using Model 2100 stimulator (A-M Systems, Carlsborg, WA). During stimulation sessions, random catch trials were interspersed (probability 1/6), during which spinal stimulation was turned off. Each period without stimulation lasted until a successful trial was performed, or for 30 s, whichever occurred first. The animal handler was not aware of when the task program injected catch trials.

In some experimental sessions, residual motor unit potentials were recorded from muscles mostly paralyzed due to the muscimol injection. In these cases, the action potential of a single motor unit could be discriminated using the same template matching technique as employed for cortical neurons.

### Data analysis

#### Assessment of muscimol paralysis efficacy

Efficacy of muscimol paralysis was assessed first by visual inspection and testing the monkeys' ability to grasp a piece of fruit. We then also compared the average EMG responses during grasp movements with and without paralysis.

#### Assessment of behavioral improvement

We measured maximum lever position during attempted trials to compare task performance between stimulation and control epochs. Since the task was self-paced, attempted trials were aligned by thresholding the firing rate of the neuron controlling stimulation. Trial epochs were 4 s long (1 s pre- and 3 s post-threshold crossing). The maximum lever position for each such epoch was determined, and the difference of mean maximum lever displacements between stimulation conditions was tested for significance using a two-tailed randomization test (*n* = 10,000; α = 0.05).

As well as lever displacement (which included successful and unsuccessful trial attempts), we also measured the rate at which animals performed successful trials (i.e., the lever was held at the target displacement for the required time and received a reward). We divided the number of successful trials completed during stimulation and control periods by the total time of each period. In order to determine the significance of differences in trial rates between stimulation and control periods, we created surrogate shuffled data by randomly allocating each trial into stimulation or control periods. To determine the length of an individual trial, we chose the time point half-way between two consecutive trial completion events as the trial boundary. We then performed a permutation test (*n* = 100,000; α = 0.05) on the shuffled trial assignments, using the difference of trial rates as the test statistic.

## Results

### Dataset

This study comprises six muscimol sessions with monkey B, spread over 18 days, and 27 sessions with monkey R, spanning 104 days. Of the sessions with monkey R, 25 employed cortical control of stimulation while one used motor unit potentials from the muscles, and another one used both. On average, monkey B performed 96 (SEM 27) trials per muscimol session and 112 (31) trials per training session. Monkey R performed 122 (18) trials per muscimol session and 260 (20) trials per training session during the same period.

### Muscimol induced paralysis

After muscimol injections and while the monkeys recovered from sedation, we assessed the monkey's ability to perform reach and grasp movements by presenting pieces of food. In monkey B, hand and forearm muscles were reliably paralyzed by muscimol injections, leading to severely disabled grasps. In monkey R, despite using more muscimol, the behavioral effects were less reliable and paralysis was often strongest on intrinsic hand muscles. In both monkeys, the effects persisted throughout the duration of the stimulation session up to several hours; stimulation sessions usually lasted 1–2 h.

### Cortical control of spinal stimulation

To produce controlled and repeatable motor deficits in macaque monkeys without requiring permanent injury, we inactivated the hand representation of M1 with focal injections of the GABA-agonist muscimol (Matsumura et al., 1991; Schieber and Poliakov, 1998; Schmidlin et al., 2008; Figure 1B). For several hours after the injection, the monkeys were impaired at a trained task that involved grasping and pulling a handle against a resistive spring load. Despite the animals' inability to displace the handle sufficiently to receive a reward, many grasp-related neurons in PMv showed robust modulation of activity during attempted trials. In each session, the firing rate of one such neuron provided a real-time read-out of the intention to move that was transformed into a control signal to modulate the rate of spinal cord stimulation. Intraspinal microstimulation was delivered either with acute electrodes inserted via a percutaneous chamber (monkey B) or through a chronically implanted floating microelectrode array (monkey R, Figure 1C). As observed previously in anesthetized animals, neighboring stimulation electrodes tended to activate different muscles of the hand and forearm (Moritz et al., 2007), and stimulation of a single electrode close to motor threshold often activated several muscles (Figure 1D) producing functional movements such as whole-hand grasping (Zimmermann et al., 2011).

**Figure 2:**
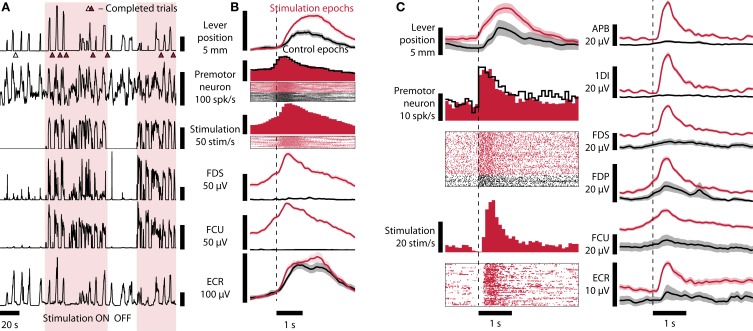
**Brain-controlled spinal stimulation improves task performance and restores muscle activity. (A)** Lever position, neural firing rate, stimulation rate, and EMG recorded from FDS, FCU, and ECR recorded during a brain-controlled stimulation session (monkey B). Consecutive stimulation epochs (shaded) and control epochs with no stimulation (no shading) are shown, incorporating several successful trials (indicated by triangles, filled: stimulation, open: control). **(B)** Average data from stimulation (124 trial attempts) and control (73 trial attempts) epochs aligned to attempt onset (inferred from neural firing rate exceeding 90 spikes/s). Raster plots show 20 stimulation and 20 control trial attempts. Shaded areas indicate standard error of the mean (SEM). Monkey B, session B100711000. **(C)** Similar to **(B)**, monkey R. Trials aligned to PMv neuron firing rate exceeding 11 Hz. One hundred twenty-nine stimulation trials and 35 control trials over a period of 29 min are shown. Shaded areas: SEM. Session Rv110719002.

**Figure 3:**
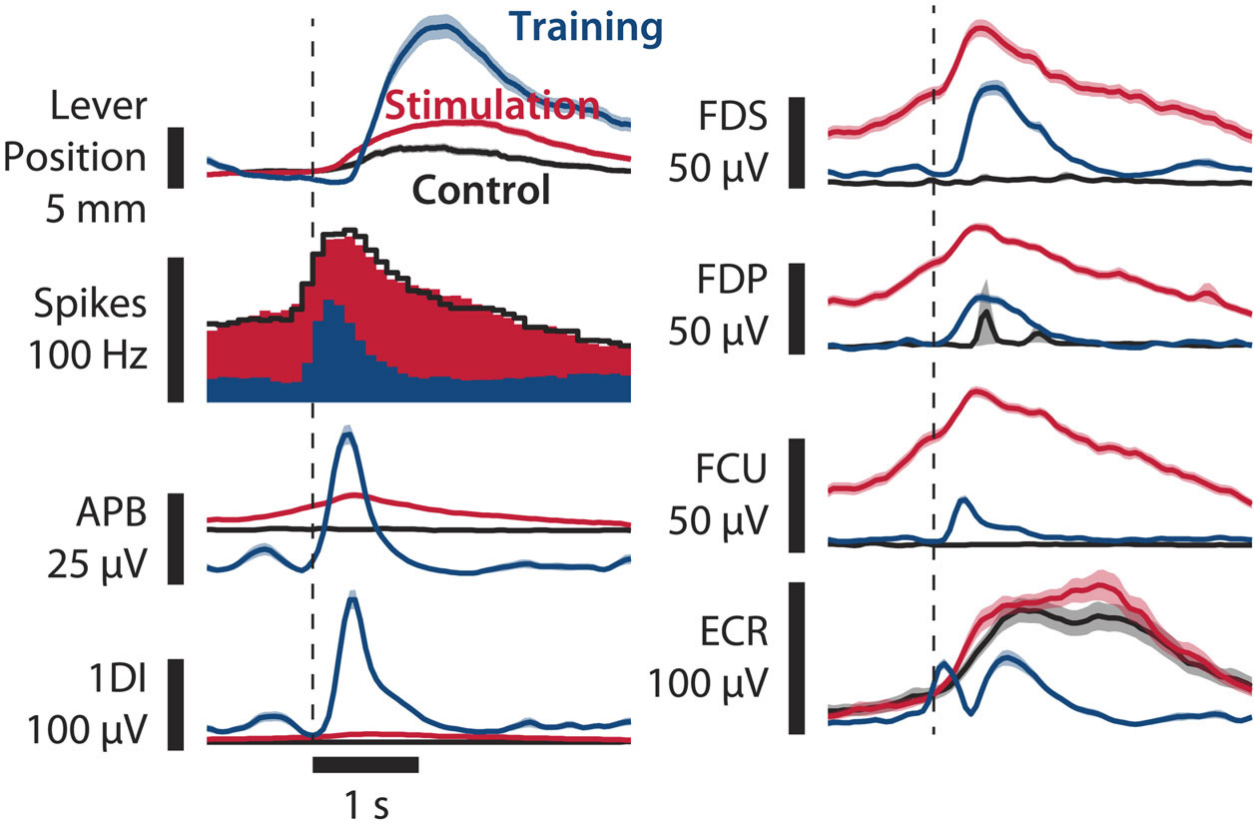
**Comparison of paralysis+stimulation, paralysis, and training trials**. Averages of lever position, neural firing, and EMG of various hand and forearm muscles are shown. Paralysis+stimulation (red, *n* = 124 trials) and paralysis (black, *n* = 73 trials) epochs are the same as in Figure 2. Training epochs (blue, *n* = 163 trials) are from a session 3 days before in which no muscimol was injected. Epochs are aligned to threshold crossing of neural firing rate (90 Hz for paralysis session, 40 Hz for training session, broken vertical line). Muscle activation due to spinal stimulation in finger flexors FDS and FDP and wrist flexor FCU resembles normal activation in shape and amplitude. Monkey B, sessions B100708001 and B100711000. Shaded areas: SEM.

Figure 2A and Supplementary Movie 1 show a typical sequence of trials incorporating periods of closed-loop stimulation interspersed with control periods during which no stimulation was delivered. Trials were aligned to the moment that the PMv firing rate crossed a firing threshold to indicate the onset of an attempted movement. Aligned in this way, firing rate profiles for trials attempted during both periods were similar (Figure 2B). However, during stimulation epochs, the monkey was able to pull the lever further and hold it longer than during control epochs (average maximum displacement of l permutation test). Moreover, during stimulation epochs, the monkey performed 3.1 successful trials/minute compared with only 1.8 successful trials/minute during control periods (mean trial lengths [SEM] 19.6 s [1.7 s] vs. 33.4 s [6.2 s], *p* = 0.03, permutation test). Trial-averaged EMG plots revealed that stimulation restored task-modulated activation profiles in muscles paralyzed by muscimol injection (Figure 2). Spinal stimulation selectively activated wrist and finger flexor muscles: little stimulation evoked activation was observed in the wrist extensor (ECR) recorded here. Comparison with data recorded during training sessions with no muscimol injection showed that EMG profiles during stimulation epochs were modulated with a time-course similar to that during natural task performance (Figure 3). The peak of activity is wider during paralysis sessions than during normal training sessions, which can be attributed both to longer durations of trials as reflected by the lever positions and variability in the control signal taken from the premotor neuron. The wrist extensor ECR was not inhibited by the muscimol block, however its activity pattern follows the slower course of the trials during paralysis sessions. On other muscles such as 1DI, the effect of spinal stimulation was rather weak compared to the activation of that muscle during normal training. Note that the baseline of APB seems elevated during both stimulation and control periods of the paralysis session. This is due to an elevated noise level affecting the channel during this recording.

In individual sessions with both animals we were able to demonstrate improvements in performance, measured both in terms of movement amplitude and rate of successful trials. In the best session with monkey R (Figure 2C), lever displacement was significantly increased (average maximum lever displacement [SEM] 14 mm [0.9 mm] vs. 6 mm [1.5 mm], *p* = 0.0001, permutation test), and was associated with a significant increase in trial completion rate during stimulation epochs (6.8 trials/min vs. 4.0 trials/min, *p* = 0.02, permutation test; average trial length [SEM] 8.9 s [0.6 s] vs. 15.0 s [3.3 s]). For comparison, the average success rates during training sessions without muscimol injection were 3.8 trials/min (monkey B) and 5.8 trials/min (monkey R).

### Control of spinal stimulation using residual muscle activity

**Figure 4:**
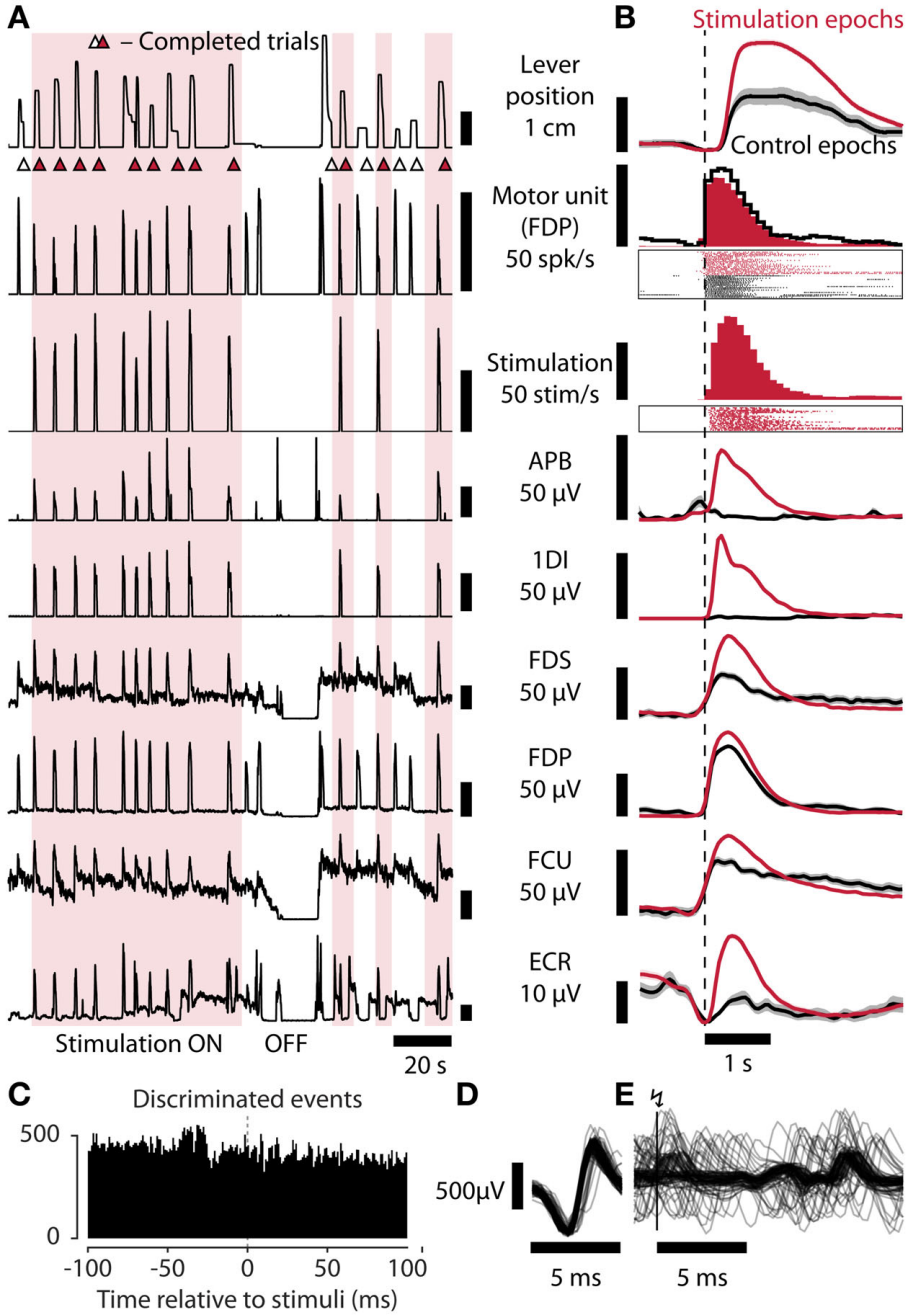
**Task performance is restored by spinal stimulation controlled by residual muscle activity. (A)** Lever position, motor unit firing rate, stimulation rate, and EMG recorded from APB, 1DI, FDS, FDP, FCU, and ECR, during a session in which residual motor unit activity from FDP was used to control stimulation (monkey R). **(B)** Average data from stimulation (178 trial attempts) and control (43 trial attempts) epochs aligned to attempt onset (inferred from motor unit firing rate exceeding 20 Hz). Shaded areas: SEM. Session Rv110714003. **(C)** Peri-Stimulus Time Histogram showing discriminated motor unit action potentials used to control stimulation relative to occurrence of stimulus pulses (0 ms). The absence of a peak following the stimulus indicates that the discriminator was neither triggered by a stimulus artifact nor by a stimulus-evoked motor response (which could lead to a positive feed-back loop). **(D)** Sample raw discriminated FDP motor unit potentials (*n* = 100). **(E)** Raw FDP EMG traces aligned to stimulation events at vertical bar (*n* = 100).

An increasing proportion of spinal cord injuries are motor incomplete (DeVivo, 2012) meaning patients retain some residual voluntary muscle control which could in principle be used to control spinal cord stimulation. Therefore, in sessions with an incomplete motor block we discriminated single motor unit activity from EMG and used this to control ISMS. In the session shown in Figure 4, the firing rate of a motor unit in the first dorsal interosseus muscle was used for online control of ISMS. Care was taken in this case to ensure that the motor unit was discriminated cleanly and that stimulation responses did not influence the control signal (Figures 4C–E). Again, average maximum lever displacement was higher with stimulation than without (20 mm [SEM 0.6 mm] vs. 12 mm [1.6 mm], *p* = 0.0001, permutation test) and trial rates were higher (6.3 trials/min vs. 3.3 trials/min, *p* = 0.007, permutation test; average trial length [SEM] 9.5 s [0.6 s] vs. 18.3 s [4.4 s]).

### Combined analysis of all sessions

The behavioral effects of muscimol injections were variable across sessions, ranging from complete paralysis of the hand to relatively minor deficits. In addition, the stimulation effects in monkey B were quite variable due to the use of acutely inserted electrodes. As a result we did not expect to obtain significant performance improvements in every session. Nevertheless, we performed a combined analysis of all sessions in which closed-loop spinal stimulation was attempted, by calculating the overall rate at which successful trials were completed when stimulation was on compared with during catch trials with no stimulation. Combining over all six recording sessions, monkey B performed a total of 476 trials in 297.3 min with stimulation, and 93 trials in 49.6 min without stimulation. This corresponds to an average rate of trial completion of 1.6 trials per minute with stimulation, and 1.9 trials per minute without stimulation. This difference that was not significant (*p* = 0.2, permutation test as before). Across 27 sessions, Monkey R performed 4671 trials in 1039.6 min with stimulation and 823 trials in 249.8 min without stimulation. This corresponded to trial completion rates of 4.5 vs. 3.3 trials per minute respectively, demonstrating a significant (*p* < 0.01) performance improvement due to stimulation across the combined sessions. That we were unable to demonstrate significance with the pooled analysis in monkey B may reflect our use in this animal of acutely positioned spinal electrodes which were less effective at producing robust, stable stimulation effects throughout every session. Nevertheless, the fact that we can demonstrate a significant benefit of spinal stimulation within individual sessions (e.g., Figures 2A,B) suggests that when electrodes were appropriately positioned, closed-loop control of spinal stimulation was effective at restoring grasp function following M1 inactivation.

### Stability of stimulation thresholds over time

**Figure 5:**
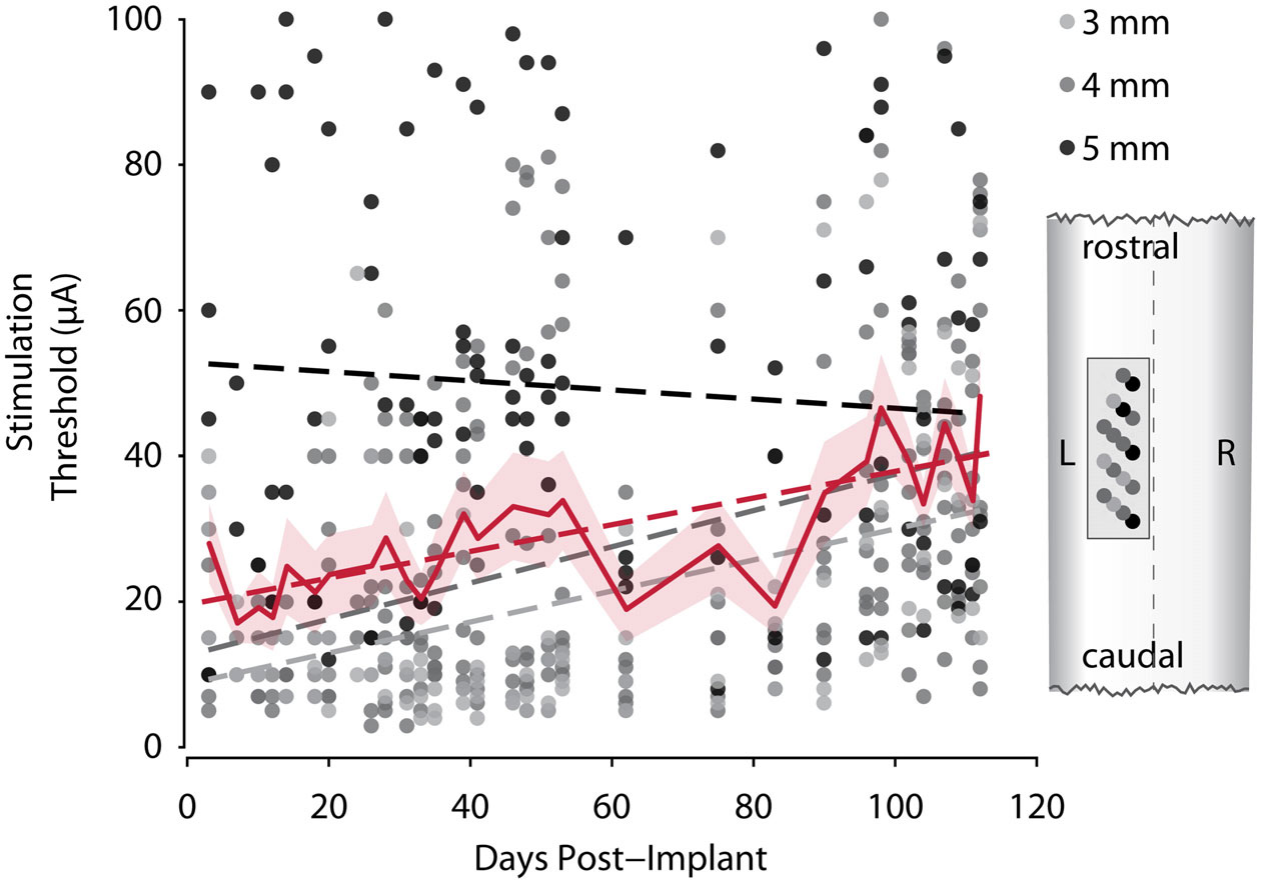
**Development of motor thresholds over time**. Dots show measured stimulation thresholds for individual electrodes of monkey R's array implant over the course of the experiment. Dots are color-coded for length of the electrode. The solid red line shows the average thresholds for each session (shaded area, SEM). Dashed lines represent linear fits for the three lengths of electrodes (gray), and all electrodes (red). The cartoon (right) shows the distribution of electrode lengths over the array and its position within the cord.

Stability of stimulation responses is a key requirement for a successful spinal implant. In monkey R we tested stimulation thresholds of all 17 electrodes on the FMA over a period from 3 to 112 days after implantation (Figure 5). Shortly after implantation, stimulation thresholds were on average around 20 μA. After 3.5 months, stimulation thresholds increased to ~40 μA on average. Individual electrodes showed considerable variation of thresholds over the duration of the experiment, and 6 of 17 electrodes had a lower threshold at the end of the experiment than just after implantation. On any given day, only a small proportion (none to 4) of the electrodes failed to respond at all to pulses of 100 μA, however even on the last day thresholds were determined, movements were evoked by stimulating each single electrode at 100 μA or below.

Grouping the electrodes by their length shows that the shorter, more lateral electrodes showed lower thresholds on average, but a larger increase in threshold over time, whereas average stimulation thresholds of longer electrodes decreased over time.

### Change of cortical signals during brain controlled ISMS

**Figure 6:**
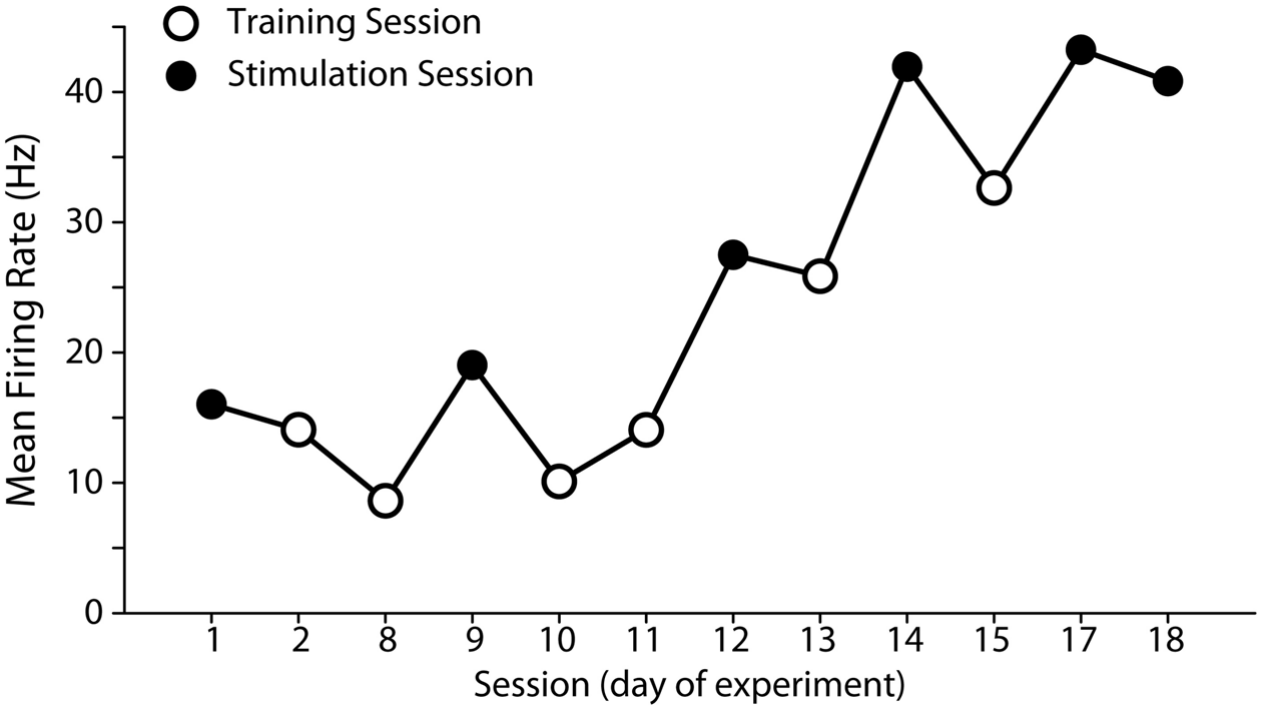
**Development of mean firing rate of the neuron used to control spinal stimulation over the course of the experiment in monkey B**. We used the same neuron to control spinal stimulation over the course of 18 days. Mean firing rate was higher during stimulation sessions (solid symbols) than in training sessions (open symbols), and increased over time (see Table 1 for linear regression statistics).

In monkey B we were able to follow the activity of one task-related PMv neuron over multiple sessions (Figure 6). During paralysis sessions this neuron was also used to control ISMS. This neuron showed a higher average firing rate during stimulation sessions compared to control sessions, which may reflect a greater volitional effort required to compensate for the motor deficits (Table 1). In addition, there was a general trend for an increase in firing rate throughout the recording period. Session length, was not found to affect the mean firing rate, however.

**Table 1:** **Multiple linear regression of mean firing rate of brain control neuron**.

**Variable**	**Coefficient**	***t*-Statistic**	***p***
Paralysis session (coded as 1—paralysis, 0—training)	9.6	3.37	0.003
Days since first experiment	1.7	5.92	1.3 × 10^−5^
Length of session	−0.001	−0.46	0.65
Overall model statistics: *F* = 20.0, *p* = 6 × 10^−6^, *R*^2^ = 0.77			

*Independent variables were session type (paralysis or training), days since first experiment, and session length*.

## Discussion

These results are to our knowledge the first demonstration of successful control of a neural prosthesis in primates after disruption of primary motor cortex. Although premotor areas such as PMv have direct projections to the spinal cord (Dum and Strick, 1991), their motor outputs are likely mediated predominantly via M1 (Schmidlin et al., 2008) consistent with the profound suppression of many muscles seen in our control epochs. In individual sessions with both animals, closed-loop control of spinal cord stimulation was successful at increasing movement amplitude and success rate. In monkey R, stable stimulation effects were facilitated by using a chronic spinal array, and a combined analysis of all sessions also revealed a significant increase in trial success rate during stimulation. Therefore, even though some residual control of muscles may have survived M1 inactivation and could have contributed to task performance, this was nevertheless assisted by the closed-loop spinal stimulation protocol we implemented. Due to reciprocal connectivity between motor and premotor cortices (Dum and Strick, 2005), inactivation of M1 might be expected to disrupt task-modulated neural activity in PMv. In fact, when we followed the same cell across multiple sessions, firing rates in PMv were higher during muscimol sessions, compared with interspersed training sessions, perhaps reflecting the increased volitional effort required to overcome the effects of M1 inactivation.

It should be noted that the reversible inactivation of M1 used in this study does not model the long-term effects of cortical or spinal cord injury, including plastic changes leading to reorganization of cortical function, up-regulation of spinal reflexes or spasticity. The success of BMI demonstrations in human patients (Hochberg et al., 2006, 2012; Collinger et al., 2013) suggests that activity related to intended movement can be decoded from the cortex long after the onset of paralysis. The efficacy of intraspinal stimulation for generating useful hand movements in human patients has yet to be similarly established. However, evidence from rats (Sunshine et al., 2013) suggests that the effects of cervical intraspinal stimulation after chronic spinal cord injury are comparable to those seen in uninjured animals. One potential advantage of relaying cortical signals directly to the spinal cord may be to reduce the maladaptive plasticity (hyperreflexia, spasticity) that occurs in a spinal cord deprived of cortical inputs, although this hypothesis is highly speculative at the current time.

Closed-loop functional electrical stimulation of muscles has previously been shown to improve performance on wrist (Moritz et al., 2008; Pohlmeyer et al., 2009) and hand tasks (Ethier et al., 2012). In addition, closed-loop local field potential control of intraspinal stimulation has been used to improve wrist function after an inadvertent injury to the spinal cord of one monkey (Nishimura et al., 2013a). Our results are the first use of cortically-controlled stimulation of the spinal cord to improve grasping function. An advantage of spinal cord stimulation is the naturalistic recruitment of multiple muscles through activation of surviving spinal circuitry (Bamford et al., 2005). In this study we were able to produce functional, whole hand grasping from stimulation at a single site, and we have previously shown that just two electrodes are required for independent control of reaching and grasping (Zimmermann et al., 2011). Extending stimulation to many electrodes should thus broaden the movement repertoire to include several grasp types. On the other hand, here we only used a simple control algorithm depending on the activity of one neuron, selected solely on the basis of task-related modulation. Grip type can be readily decoded in monkeys (Vargas-Irwin et al., 2010; Bansal et al., 2012) and humans (Pistohl et al., 2012) using intracortical arrays and electrocorticography. By combining multi-electrode stimulation with sophisticated decoding algorithms, it should therefore be possible to restore control over a range of upper-limb behaviors.

Robust responses to stimulation were observed in one animal over a 112-day period, but stimulation thresholds, particularly those of the shorter electrodes in our array, did increase slowly over the course of the experiment. This may be explained by scar tissue forming between the array and the surface of the spinal cord gradually pushing the electrodes upwards. Since spinal cord injured patients can live for many decades, the stability of electrode implants remains a major challenge for clinical applications of intraspinal microstimulation. It is possible that epidural stimulation of either the dorsal or ventral spinal cord may provide a less invasive and more stable approach for reanimating the paralyzed limb (Sharpe and Jackson, 2014).

Although we only implemented closed-loop stimulation during experimental sessions, emerging evidence suggests long-term, continuous use of neural prostheses may have further applications in neurorehabilitation by driving activity-dependent plastic changes (Jackson and Zimmermann, 2012). Jackson et al. (2006) demonstrated in healthy monkeys that closed-loop cortical stimulation could strengthen cortical connectivity by a Hebbian process. Recently, Guggenmos et al. (2013) have extended this idea to a rodent model of focal brain injury, demonstrating enhanced functional connectivity and lasting improvements to grasp function using a neural prosthesis that connected premotor and somatosensory cortex. Finally, Nishimura et al. (2013b) have demonstrated that similar Hebbian mechanisms may also act at corticospinal connections. We can therefore speculate that the causal correlations between cortical and spinal activity introduced by long-term closed-loop stimulation may have further benefits for patients with incomplete injuries by strengthening surviving descending pathways.

### Conflict of interest statement

The authors declare that the research was conducted in the absence of any commercial or financial relationships that could be construed as a potential conflict of interest.
